# A System for Performing High Throughput Assays of Synaptic Function

**DOI:** 10.1371/journal.pone.0025999

**Published:** 2011-10-05

**Authors:** Chris M. Hempel, Michael Sivula, Jonathan M. Levenson, David M. Rose, Bing Li, Ana C. Sirianni, Eva Xia, Timothy A. Ryan, David J. Gerber, Jeffrey R. Cottrell

**Affiliations:** 1 Galenea Corporation, Cambridge, Massachusetts, United States of America; 2 Department of Biochemistry, Weill Cornell Medical College, New York, New York, United States of America; University of Houston, United States of America

## Abstract

Unbiased, high-throughput screening has proven invaluable for dissecting complex biological processes. Application of this general approach to synaptic function would have a major impact on neuroscience research and drug discovery. However, existing techniques for studying synaptic physiology are labor intensive and low-throughput. Here, we describe a new high-throughput technology for performing assays of synaptic function in primary neurons cultured in microtiter plates. We show that this system can perform 96 synaptic vesicle cycling assays in parallel with high sensitivity, precision, uniformity, and reproducibility and can detect modulators of presynaptic function. By screening libraries of pharmacologically defined compounds on rat forebrain cultures, we have used this system to identify novel effects of compounds on specific aspects of presynaptic function. As a system for unbiased compound as well as genomic screening, this technology has significant applications for basic neuroscience research and for the discovery of novel, mechanism-based treatments for central nervous system disorders.

## Introduction

The application of unbiased, high-throughput screening approaches has had a major impact on basic research and drug discovery. For example, forward genetic screens in yeast and flies have yielded fundamental insights into a variety of complex biological processes [1], [2], microarray-based screens have provided a comprehensive means to examine regulation of gene expression [3], and small molecule library screens have been critical for identifying chemical modulators of biological processes for drug discovery applications [4]. Despite the extensive body of research focused on synaptic mechanisms in mammalian neurons, there are no screening tools capable of performing dynamic measurements of synaptic activity in a high-throughput format. Such tools would enable the performance of genetic and pharmacological screens to comprehensively examine the molecular biology of the synapse and to identify novel modulators of synaptic function. Moreover, since altered synaptic function has been associated with a number of psychiatric and neurological disorders [5], [6], [7], [8], [9], [10], [11], [12], [13], [14], the identification of novel proteins or compounds that modulate or restore aberrant synaptic function involved with disease pathogenesis is an attractive approach for the discovery of new mechanism-based therapies.

More specifically, an alteration of synaptic vesicle cycling has been implicated in a variety of disorders, including schizophrenia, Alzheimer's disease, Parkinson's disease, Huntington's disease, and epilepsy [5], [6], [7], [8], [9], [10], [11], [12], [13], [14]. A fundamental component of synaptic transmission, synaptic vesicle cycling is a complex, multi-stage process that includes the steps of vesicle exocytosis, endocytosis, reinsertion into the recycling pool, mobilization to the active site, and priming for a subsequent round of exocytosis [15]. In recent years, powerful methods utilizing fluorescent reporters have emerged for monitoring presynaptic function in living neurons [16]. These assays are typically performed on a fluorescence microscope to image the effects of physiologically relevant patterns of action potentials, elicited by an integrated field stimulation system, on reporters of presynaptic activity [17]. However, since these methods are time and labor intensive, they are not amenable to unbiased screening applications.

The translation of presynaptic assays into a high-throughput screening system is technically challenging largely due to a requirement for long-term kinetic measurements (>5 min/well) [17]. To achieve an acceptable throughput, a presynaptic screening system must perform assays in all wells of a 96-well plate in parallel. This need for parallelization imposes significant technical demands on the imaging and stimulation components of the technology. For example, high-content imaging systems have single-synapse resolution and sufficient optical sensitivity, but they are limited to measuring one well at a time [18]. In contrast, plate readers capable of performing 96 parallel kinetic fluorescence measurements have significantly reduced optical sensitivity. Therefore, a presynaptic screening technology requires a parallel imaging system with excellent optical sensitivity and a reporter system that yields high signal density and signal-to-background properties. Finally, this assay parallelization requires the integration of an electrode array that can deliver field potentials simultaneously to neurons in all wells of 96-well plates such that all neurons are exposed to uniform current densities.

Here, we report the development of the MANTRA™ (Multiwell Automated NeuroTRansmission Assay) system: a high-throughput screening technology for performing assays of the synaptic vesicle cycle in primary neurons. We demonstrate that this system can perform 96 parallel synaptic vesicle cycle assays, meets the critical technical specifications necessary to carry out unbiased, functional screening of presynaptic neuronal function, and is capable of rapidly identifying novel effects of test agents on synaptic vesicle cycling. Through its current application and its capacity to be extended to additional synaptic processes, this technology has tremendous potential for broadening our understanding of synaptic function and, ultimately, developing new classes of mechanism-based treatments for the many diseases associated with synaptic dysfunction.

## Results

### MANTRA system development

A high throughput screening technology for monitoring synaptic vesicle cycling requires four technology components: a reporter system, an imaging system, a field stimulation system, and a data analysis system. We initiated technology development by evaluating potential solutions for these components.

**Figure 1:**
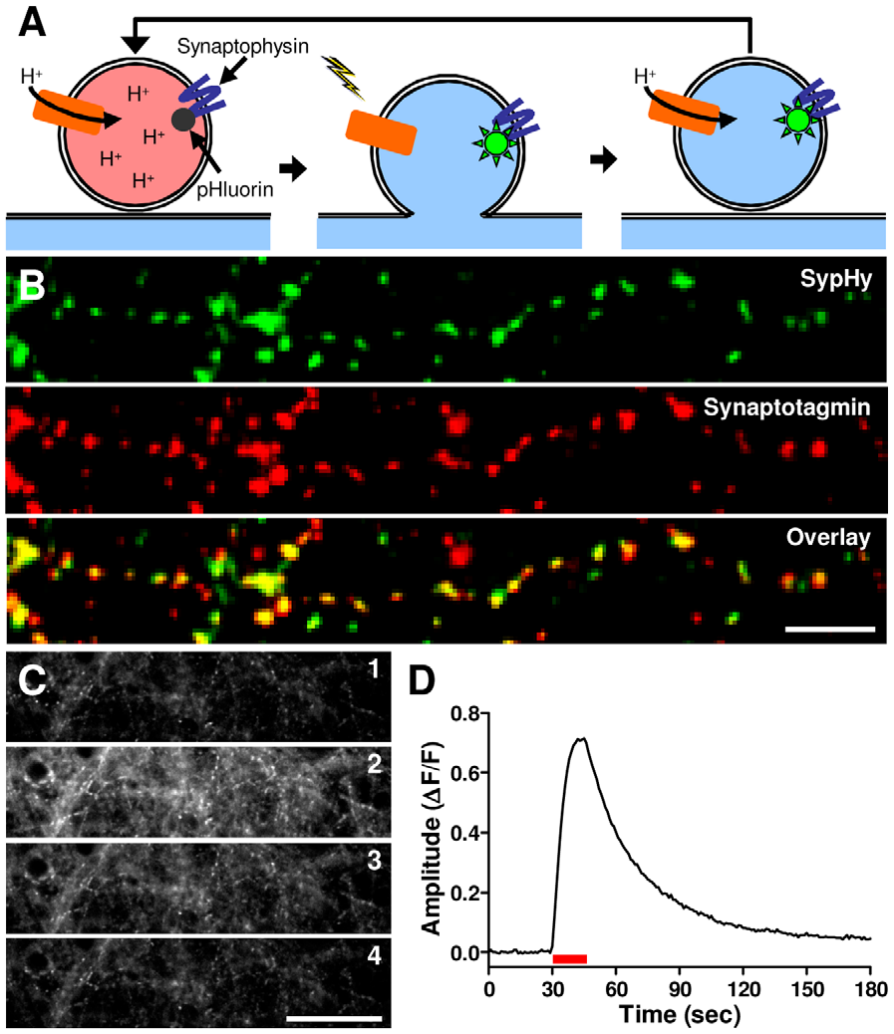
SypHy delivered by AAV transduction as a reporter of presynaptic function. (**A**) Schematic illustrating the function of the sypHy reporter of synaptic vesicle cycling. (**B**) Presynaptic localization of sypHy in neuronal cultures infected with hSyn-sypHy-AAV at 7 days *in vitro* (DIV) and fixed at 22 DIV shown by colocalization of anti-GFP (green) and anti-synaptotagmin I (red) immunoreactivity. Scale bar: 10 µm. (**C**) Portion of a kinetic fluorescence image series of an hSyn-sypHy-AAV infected culture prior to stimulation (1), during delivery of a 50 Hz, 10 second stimulus train (2), 30 sec after offset of stimulus train (3), and 2 min after offset of stimulus train (4). Scale bar: 50 µm. (**D**) Relative fluorescence intensity of the entire imaging field from the experiment shown in (c). Red bar indicates the stimulus.

We chose a pHluorin-based protein as the reporter technology because it is genetically encoded, enabling homogeneous assays [19], can report both vesicle exocytosis and endocytosis [20], and can report repeated rounds of vesicle cycling [20]. This reporter system is based on the fusion of a pH-sensitive GFP variant, pHluorin, to the lumenal domain of a transmembrane synaptic vesicle protein (Figure 1A). At the internal pH of a resting vesicle, the pHluorin fluorescence is quenched. When action potentials are elicited in neurons expressing the reporter, vesicles exocytose, exposing the pHluorin to the neutral pH of the synaptic cleft causing an increase in its fluorescence. Upon endocytosis and reacidification of the vesicles, fluorescence is again quenched. Due to its reported excellent signal-to-background properties, we opted for a synaptophysin-pHluorin fusion construct (sypHy) in which pHluorin is inserted into the second intralumenal loop of synaptophysin [21].

To maximize the number of neurons expressing the reporter, we used an adeno-associated virus (AAV) delivery system that yields a high infection efficiency with minimal cytotoxicity [22]. To eliminate non-neuronal expression, we utilized the human synapsin promoter [23] to drive reporter expression. Immunohistochemistry performed on neurons infected with this virus (hSyn-sypHy-AAV) demonstrated that sypHy is expressed in a punctate pattern that colocalizes with synaptotagmin I, indicating that it is targeted to presynaptic terminals (Figure 1B). Colocalization analysis showed that most terminals contained detectable sypHy levels (mean ± SEM: 80±3% synapses; n = 3 cultures). Using single-channel, high resolution microscopy [17], we verified that this reporter system is capable of quantifying the synaptic vesicle cycle in primary neurons (Figure 1C,D).

Since synaptic vesicle cycling assays are of long duration, screening must be performed in 96 wells in parallel to achieve an acceptable throughput. In addition, due to the lack of amplification of the reporter signal, the instrument must have highly sensitive optics. For the imaging component of the MANTRA system, we selected the plate::vision plate reader (Perkin Elmer) based on its parallel, high sensitivity imaging capacity. The plate::vision measures fluorescence in a 500 µm wide segment of each of 96 wells simultaneously and with high sensitivity by employing a unique 96-minilens array and an intensified charge-coupled device (CCD) camera [24]. Critically, assay plates in this instrument are readily accessible during imaging, permitting integration of a stimulation system.

Delivering uniform electrical stimuli to neuronal cultures in 96-well plates is a major technical challenge. To achieve acceptable assay variability, field stimuli must be spatially uniform across the neurons within each well of the entire plate, which requires carefully shaped and precisely positioned electrodes. Shape and positioning must match precisely from well to well despite considerable variability in the plate manufacturing process. For these reasons, traditional field stimulation electrodes, such as paired tungsten filaments or glass pipettes, are insufficient for the task. Therefore, for the MANTRA stimulation system, we identified and optimized an existing automated electroporation system (CellaxessHT; Cellectricon) that solved these technical hurdles as described below.

**Figure 2:**
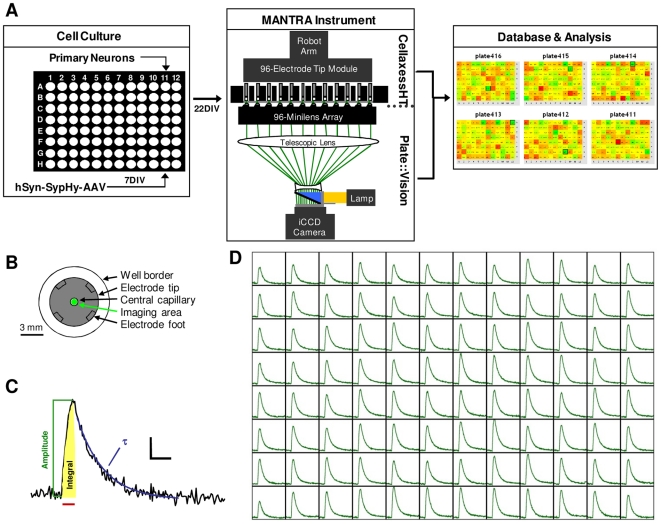
The MANTRA system. (**A**) Schematic depicting the MANTRA system, including the cell culture system, the reporter system, the instrumentation, and the heatmap application of the data analysis system. (**B**) Schematic depicting the relative dimensions of an electrode tip and the imaging area within a single well of a 96-well plate. (**C**) Representative sypHy fluorescence trace from a single well when stimulated as in (D) showing three of the basic waveform features automatically extracted by the MANTRA system analysis software. Red bar indicates stimulus. Scale bar: 0.05 ΔF/F, 20 sec. (**D**) Representative dataset from the MANTRA system showing sypHy fluorescence responses to a 50 Hz, 15 sec stimulus train from all wells of a 96-well plate. For each well, y-axis: 0.35 ΔF/F, x-axis: 180 sec.

We constructed the MANTRA instrumentation by integrating a plate::vision plate reader with a customized CellaxessHT system within a temperature controlled cabinet (Figure 2A). In typical use, a modified liquid handling unit with a robotic arm places the electrode module into the assay plate. The electrodes each consist of two concentric titanium tubes separated by a polytrifluorochloroethylene (PTFCE) tip that serves as an electrical insulator and the contact with the well bottom. The combination of force exerted by the robot arm, an internal spring mechanism, and four 75 µm high feet on the bottom of each tip ensure that all electrodes are at a uniform height (Figure 2B). In this position, the outer titanium tube is submerged in assay buffer. Liquid handling pistons draw buffer into the inner titanium tubes to create an electrical contact between the electrodes. The plate::vision initiates kinetic fluorescence sampling, and field stimulation pulses are then applied by a pulse generator. Uniform electric fields are achieved within individual wells through specific contouring of the bottom of the PTFCE electrode tip. All stimulation, imaging, and tip washing activities are coordinated by CellaxessHT control software (Cellectricon).

The MANTRA system generates 96 channels of complex waveform data. Multiple features of these waveforms yield information about different aspects of the synaptic vesicle cycle [25] (Figure 2C). To enable efficient analysis of these complex data, we created a data handling system with which pulse train information, raw fluorescence traces, extracted waveform features, and treatment conditions are automatically loaded into a relational database and processed, permitting visualization and further analysis through a web-based user interface (Figure 2A). In a typical dataset, a sypHy fluorescence response to a field stimulation train is observed in all wells of a 96-well plate (Figure 2D), demonstrating the capacity of the MANTRA system to perform 96 parallel synaptic vesicle cycling assays.

### MANTRA system technical performance

**Figure 3:**
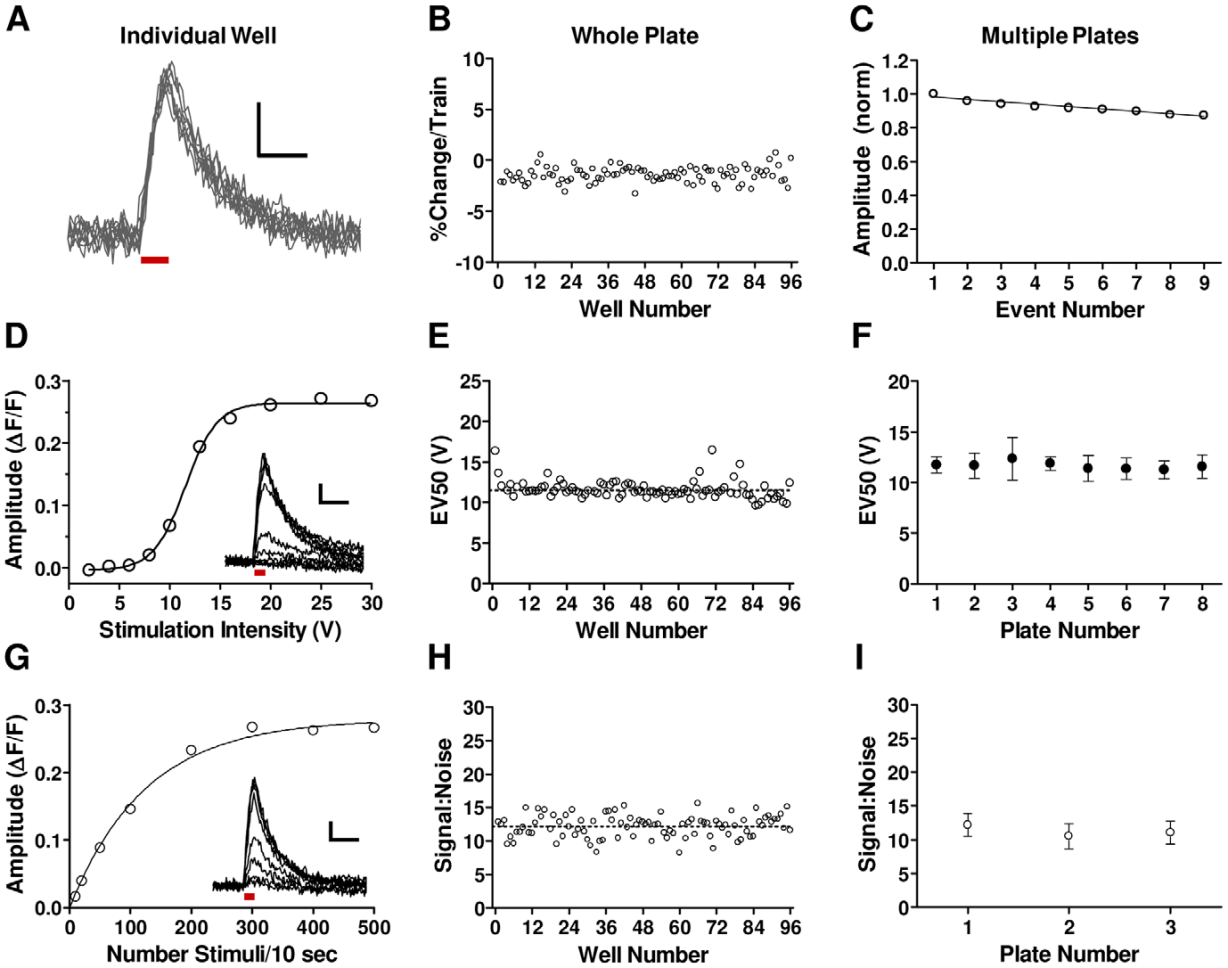
Validation of the MANTRA system technical performance. (**A**) Superimposed traces from nine successive 10 Hz, 10 sec trains from a single well. Bar indicates stimulus. Scale bars: 0.05 ΔF/F, 20 sec. (**B**) Amplitudes from all wells of a plate were calculated for the nine trains and normalized to the first train. Shown is the mean percent change per train for each well. Amplitude change per train for all wells was −1.5±0.97% (mean ± SD). (**C**) Normalized amplitudes (mean ± SD) to each train for three plates. Data were fit by linear regression (slope = −0.014; r^2^ = 0.93; p<0.0001). Amplitude change per train across plates was −1.5±0.13% (mean ± SEM; n = 3). (**D**) Amplitudes of responses to 30 Hz, 10 sec trains delivered at increasing voltages from a representative well. Data were fit with a sigmoid function (R^2^ = 0.99). Inset: Individual traces. Scale bar: 0.05 ΔF/F, 30 sec. Bar indicates stimulus. (**E**) The voltage generating the 50% peak response (EV_50_) for each well of a plate (%CV = 9.8%). (**F**) The EV50 (mean ± SD) from all wells of multiple plates stimulated as in (e). (**G**) Amplitudes from a well stimulated with 10 sec trains of increasing frequencies. Data were fit with a one-phase exponential curve (R^2^ = 0.99). Inset: Individual traces. Scale bar: 0.05 ΔF/F, 30 sec. Bar indicates the stimulation. (**H**) Signal:noise for responses to the 50 pulse train (mean ± SD: 12.2±1.7) for a plate. “Signal” is amplitude. “Noise” is the standard deviation of a 10 second baseline. (**I**) Signal:noise (mean ± SD) data generated from a 50 pulse train from three plates.

We undertook a series of studies to characterize the performance of the MANTRA system in relation to the crucial performance criteria necessary for a robust screening platform. The ability to measure responses to multiple rounds of stimulation is essential for monitoring presynaptic activity under a variety of activity regimes. We exposed hSyn-sypHy-AAV infected neuronal cultures in 96-well plates to repeated stimulus trains and observed that sypHy responses were stable over time, changing in amplitude by less than 2% per train on average (Figure 3A-C). Thus, the MANTRA system can be used to repeatedly stimulate neuronal cultures and consistently measure sypHy responses with minimal bleaching or acute cytotoxicity.

To achieve uniformity of presynaptic responses, the field stimuli applied to neurons must have a high degree of well-to-well and plate-to-plate uniformity. We examined sypHy responses to stimulus trains delivered at increasing voltages and observed a sigmoid relationship between voltage and response amplitude (Figure 3D), from which we could calculate an EV_50_ value for each well (see Figure 3E legend). EV_50_'s were highly consistent within and across plates (Figure 3E, F), demonstrating that the field stimulation system consistently delivers uniform stimuli.

Analysis of the optical sensitivity of the MANTRA system showed that it can consistently detect sypHy responses to as few as 50 stimuli within wells of a single plate and across plates (Figure 3G–I), enabling use of a large range of stimulus regimes that place different demands on the synaptic vesicle cycling machinery. To quantify well-to-well and plate-to-plate variability of sypHy responses to screening stimuli, we subjected eight plates to a protocol consisting of three pulse trains: 5 Hz, 30 seconds; 10 Hz, 30 seconds; and 50 Hz, 15 seconds. Within-plate and between-plate summary statistics for extracted parameters from the responses to 10 Hz trains are shown in Table 1 and from the 5 and 50 Hz trains in Tables S1 and S2, respectively. %CV's across all measures were between 10 and 18%, indicating the achievement of assay uniformity appropriate for screening applications.

**Table 1:** MANTRA system signal uniformity analysis.

		Individual Plates								All Plates		
Parameter		1	2	3	4	5	6	7	8	Mean	SD	%CV
Amplitude(ΔF/F)	Mean	0.16	0.15	0.15	0.19	0.15	0.17	0.16	0.17	0.16	0.02	9.0
	SD	0.02	0.02	0.02	0.02	0.02	0.03	0.03	0.03			
	%CV	13.2	11.6	12.7	10.1	15.6	17.2	15.5	15.0	13.9		
Decay τ(sec)	Mean	15.5	13.3	13.0	16.5	13.0	17.7	16.5	16.6	15.2	1.88	12.3
	SD	1.54	2.02	1.91	2.19	1.89	3.17	1.88	2.22			
	%CV	9.9	15.2	14.7	13.3	14.5	18.0	11.4	13.4	13.8		
Derivative(ΔF/sec)	Mean	0.019	0.018	0.019	0.020	0.017	0.020	0.020	0.020	0.019	0.001	5.5
	SD	0.003	0.003	0.002	0.003	0.003	0.004	0.004	0.003			
	%CV	17.0	16.9	12.8	16.4	15.0	17.8	18.6	14.8	16.1		
Integral(ΔF/F x sec)	Mean	3.33	3.27	3.31	4.04	3.46	3.59	3.51	3.74	3.53	0.261	7.4
	SD	0.41	0.41	0.43	0.43	0.53	0.59	0.49	0.55			
	%CV	12.2	12.4	13.1	10.7	15.3	16.5	14.0	14.6	13.6		
Noise (ΔF/F)	Mean	0.007	0.006	0.006	0.006	0.004	0.008	0.006	0.007	0.006	0.001	
	SD	0.001	0.001	0.001	0.001	0.001	0.001	0.001	0.001			

Eight control plates were subjected to a stimulus protocol comprised of 1) a 5 Hz, 30 sec, 2) a 10 Hz, 30 sec, and 3) a 50 Hz, 15 sec pulse train in succession, with a 5 minute inter-train interval. Amplitude, decay time constant, peak first derivative, response integral, and baseline noise for the response to the 10 Hz train are shown.

**Figure 4:**
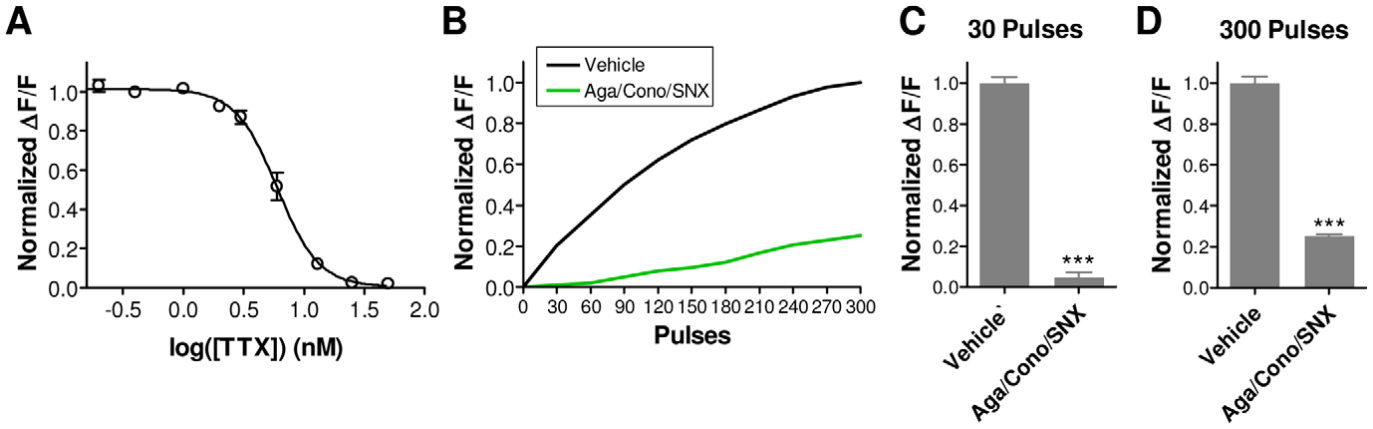
MANTRA system responses depend on action potential-mediated opening of presynaptic Ca^++^ channels. (**A**) TTX concentration-response curve for response amplitudes to a 30 Hz, 10 sec pulse train from a single plate. Each point shows the mean ± SEM of 8 wells normalized to within-plate vehicle controls. Data were fit with a standard sigmoid concentration-response function (R^2^ = 0.99). (**B**) SypHy fluorescence responses during a 30 Hz, 10 second stimulus train in the presence of the Cav2.1 inhibitor ω-agatoxin IVA (500 nM), the Cav2.2 inhibitor ω-conotoxin GVIA (1 µM), and the Cav2.3 inhibitor SNX-482 (1.2 µM). The waveforms depicted are an average of 24 wells for the vehicle and 8 wells for the treatment group. (**C,D**) Amplitudes (mean ± SEM) of the sypHy responses shown in (B) following (C) 30 pulses (1 sec) and (D) 300 pulses (10 sec) of the stimulus train (***: p<0.0001).

We next confirmed that the MANTRA system induces and measures synaptic vesicle cycling in response to action potential-mediated opening of presynaptic Ca^++^ channels. First, we found that tetrodotoxin (TTX), an inhibitor of the voltage-gated sodium channels that carry action potentials [26], potently blocked the fluorescence response to field stimulation (Figure 4A; IC_50_ = 4.9±0.6 nM; n = 3 plates), demonstrating that action potentials are required for the sypHy signal in the MANTRA system. Second, since synaptic vesicle fusion is triggered primarily by Ca^++^ influx through channels from the Cav2 family [27], [28], we analyzed the effects of inhibitors of these channels on the MANTRA system. We used ω-agatoxin IVA, ω-conotoxin GVIA, and SNX-482 for blockade of Cav2.1, Cav2.2, and Cav2.3, respectively [29], [30], [31]. Figure 4B shows the sypHy responses during a 30 Hz, 10 second stimulus train in the presence of these blockers. Early in the train (30 stimuli), Cav2 channel blockade reduced the sypHy response by >95% (Figure 4C; t-test: p = 10^−17^; n = 8). At the end of the train (300 stimuli), this treatment resulted in a >75% inhibition of the response (Figure 4D; t-test: p = 10^−18^; n = 8). These data show that the sypHy responses of the MANTRA system are dependent upon action potential-mediated opening of presynaptic voltage-gated Ca^++^ channels, consistent with typical synaptic vesicle release. The reduction in the block of fluorescence responses with increasing stimulation is likely due to action potential broadening following Cav2 channel inhibition [32] and an increasing amount of Ca^++^ influx from other sources, such as T-type or L-type Ca^++^ channels [33], [34].

Since the synaptic vesicle cycle is sensitive to temperature fluctuations [35], a thermostat system was incorporated into the MANTRA instrumentation. Testing under typical use conditions showed that this system can maintain the temperature of assay wells to within a range of ±0.5°C (Figure S1). In summary, these data demonstrate that the MANTRA system is capable of performing 96-parallel synaptic vesicle cycling assays with high precision, uniformity, sensitivity, and reproducibility.

### MANTRA system can identify synaptic vesicle cycling modulators

**Figure 5:**
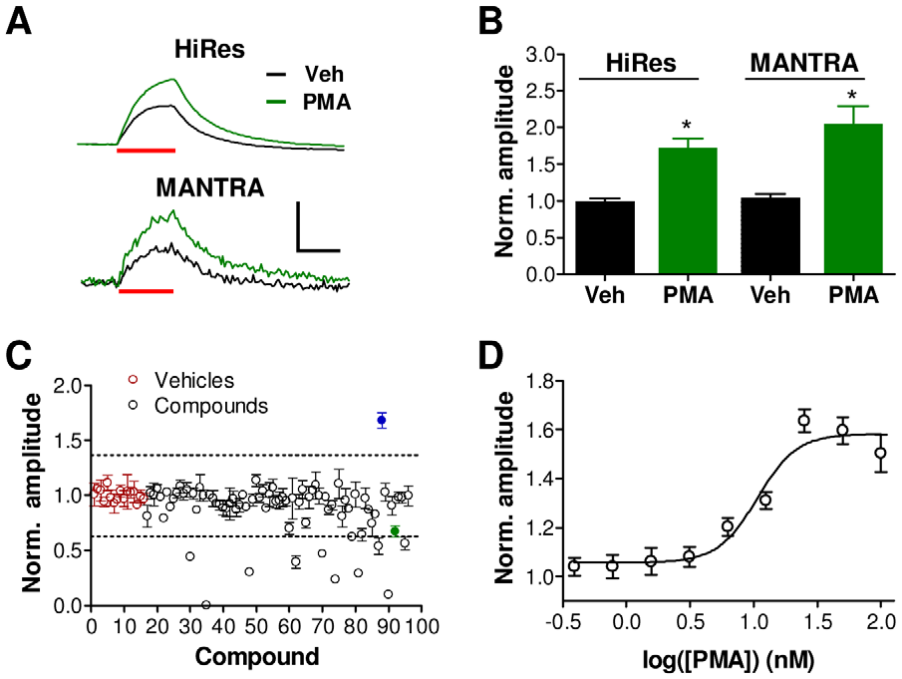
The MANTRA system can detect modulators of synaptic vesicle cycling. (**A**) SypHy traces from neuronal cultures treated with PMA (1 µM) or vehicle (0.7% DMSO) generated with the high resolution microscope system (HiRes) or the MANTRA system in response to a 5 Hz, 30 sec stimulus train. Scale bar: 0.1 ΔF/F, 20 sec. (**B**) Vehicle-normalized amplitudes (mean ± SEM) in response to the 5 Hz, 30 sec stimulus train in vehicle- and PMA-treated cultures measured on the microscope based system (HiRes; n = 3) and on the MANTRA system, for which statistics were generated from three randomly selected PMA- and vehicle-treated replicates. (**C**) The plate of the LOPAC library containing PMA was screened in triplicate on the MANTRA system (10 µM; 1 hour incubation) using a stimulation protocol consisting of a 5 Hz, 30 sec train, a 10 Hz, 30 sec train, and a 30 Hz, 15 sec train with stimulation trains separated by 5 minute intervals. Shown are the amplitudes (mean ± SEM) of the three replicates for each compound normalized to the mean amplitude of the vehicle wells. Dotted lines indicate three standard deviations from the vehicle mean. Red points indicate vehicle wells, blue point indicates PMA. Green point indicates N6-phenyladenosine as further described in Figure 6. (**D**) PMA concentration-response curve for response amplitudes to the 5 Hz train generated from a single plate. Each point shows the mean ± SEM of 8 wells normalized to within-plate vehicle controls. Data were fit with a standard sigmoid concentration-response function (R^2^ = 0.95).

To evaluate its utility for high-throughput screening, we examined whether the MANTRA system can detect modulators of synaptic vesicle cycling. The phorbol ester phorbol–12-myristate-13-acetate (PMA) has been shown to enhance synaptic vesicle release [36]. Using our microscope-based, high-resolution system, we found that application of PMA (1 µM) increased the amplitude of sypHy responses to a 5 Hz, 30 sec stimulus train (Figure 5A,B; t-test: p = 0.004; n = 3). We next examined the ability of the MANTRA system to detect this compound-induced alteration in synaptic vesicle cycling. PMA (1 µM) was added to multiple wells of a 96-well plate, which was then subjected to the same stimulation protocol on the MANTRA instrument. The amplitudes of the fluorescence responses to the stimulus trains from a set of three randomly selected PMA wells were significantly greater than those of three randomly selected control wells (Figure 5A,B; t-test: p = 0.01). These results recapitulate the effects of PMA observed using the high resolution system and confirm the ability of the MANTRA system to measure the effects of synaptic vesicle cycling modulators.

To demonstrate that the MANTRA system can identify modulators of synaptic vesicle cycling in high throughput screening mode, we screened the plate of the Library of Pharmacologically Active Compounds (LOPAC; Sigma) that contains PMA. Given the response variability documented in Table 1, compounds were screened in triplicate to improve sensitivity to modest compound effects. Each of three assay plates contained a single copy of each compound assigned to a pseudo-randomized well location. Neurons were stimulated with three pulse trains that varied in frequency (5 Hz, 30 sec; 10 Hz, 30 sec; 30 Hz, 15 sec). Using a hit detection threshold set at three standard deviations from the mean of the control well values, PMA was detected as a hit that increased the amplitude of the response to the 5 Hz train (Figure 5C; mean ± SEM standard score: 5.6±0.56). Eleven additional hits were identified that reduced the response to the 5 Hz train (see Dataset S1 for complete dataset). Each of these compound effects was confirmed on a separate set of hit confirmation plates (Figure S2).

We next generated a concentration-response curve for the effects of PMA on the amplitude of sypHy responses to 5 Hz stimulus trains using a single assay plate (Figure 5D). The EC_50_ for the PMA-induced increase in sypHy response amplitude was 6.2±2.4 nM (mean ± SEM; n = 3 plates). The ability to rapidly identify modulators of synaptic vesicle cycling and to generate a concentration-response curve from a single plate demonstrates the utility of the MANTRA system for screening applications.

### Identification of a presynaptic modulatory mechanism

**Figure 6:**
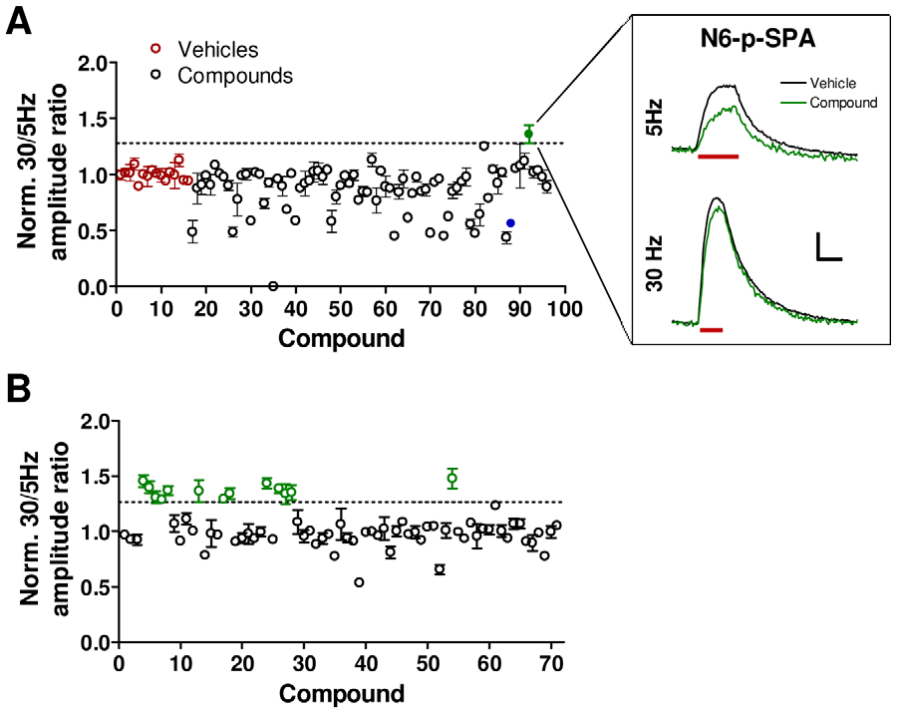
High frequency stimulation overcomes adenosine A1 agonist-induced suppression of synaptic vesicle release. (**A**) The ratio of the amplitudes of the responses to the 30 Hz train to amplitudes of the responses to the 5 Hz train was determined for the compounds from the LOPAC plate described in Figure 5C. The depicted data (mean ± SEM) were normalized to the mean of the vehicle control wells. Dashed line indicates three standard deviations from the mean of the vehicles. Red points indicate vehicle wells. Blue point indicates PMA. Green point indicates N6-phenyladenosine. Inset shows the average waveforms from the three replicates of N6-phenyladenosine and the 36 vehicle replicates from the three replicate screening plates. Red bars indicate periods of stimulation Scale bar: 0.05 ΔF/F, 20 sec. (**B**) A single plate of compounds targeting adenosine and purine receptors (Enzo Life Sciences) was screened as described in Figure 5C. Data are as described in (A). Green circles indicate compounds that generated effects greater than three standard deviations from the mean of the vehicles.

The MANTRA system generates a rich dataset regarding the effects of compounds on multiple aspects of presynaptic function under different activity regimes. Further analysis of the LOPAC plate data described above revealed specific effects of compounds on synaptic vesicle cycling induced by the different stimulation intensities. In particular, the adenosine A1 receptor agonist N6-phenyladenosine is the only compound on the plate that decreased the amplitude of responses to 5 Hz stimulation (see Figure 5C) with little or no effect on responses to 30 Hz stimulation (Figure 6A; mean ± SEM 30 Hz/5 Hz amplitude ratio standard score: 3.8±0.86). To determine if this effect is common to compounds with the same pharmacological activity, we screened a plate of 71 compounds consisting of modulators of adenosine or purine receptors (Enzo Life Sciences). We found 13 hit compounds that increased the 30 Hz/5 Hz amplitude ratio, and 11 of these hits are known adenosine A1 agonists (Figure 6B; see Dataset S2 for complete dataset). In all cases, the increase in this ratio resulted from a decrease in the amplitude of the response to the 5 Hz train and not from an increase in the 30 Hz response amplitude (Figure S3). Adenosine A1 receptors have long been known to be presynaptic receptors whose activation results in reduced synaptic vesicle release [37], [38]. Our screening results suggest that high frequency stimulation can overcome this inhibitory mechanism and demonstrate the capacity of the MANTRA system to rapidly identify novel mechanistic aspects of synaptic vesicle cycling.

## Discussion

In this report, we describe the development and validation of the MANTRA system: a high throughput technology capable of performing kinetic assays of the synaptic vesicle cycle directly in primary neurons. We demonstrate that this system operates with high precision, uniformity, sensitivity, and reproducibility and is capable of detecting modulators of synaptic vesicle cycling in a high-throughput screening mode. When using a complex stimulation protocol covering a broad range of synaptic activity regimes, at least 1,500 wells, or 500 agents when screened in triplicate, can be analyzed on the MANTRA system per day. With this throughput, the system can be used to screen for modulators of presynaptic function.

Two key technological advances were necessary for the development of the MANTRA system: 1) a primary neuronal culture and synaptic vesicle cycling reporter system that generates strong signals with a high signal-to-background ratio, and 2) novel instrumentation integrating a sensitive, parallel-imaging component and a 96-electrode stimulation system. While other screening instruments with integrated imaging and field stimulation components have been described, they are limited to measuring eight wells in parallel [39], [40]. Since those systems were developed for ion channel assays, which are typically of short duration, this limitation has a relatively minor impact on assay throughput in that context. However, for analysis of the synaptic vesicle cycle, assay durations of tens of minutes are required to cover a broad range of stimulation parameters. Therefore, the throughput of the previously described instruments [39], [40] is insufficient for high-throughput screening of presynaptic function.

### Potential impact of the MANTRA system

The MANTRA system enables unbiased functional high throughput screening of presynaptic activity directly in primary neurons using physiologically relevant patterns of stimulation. This application has the potential to significantly expand our understanding of presynaptic function. As described in this report, the MANTRA system can be used for screening libraries of chemical agents with known pharmacological activities to elucidate presynaptic molecular mechanisms. By employing this approach and screening two compound plates, we were able to rapidly identify a novel property of adenosine A1 receptor-mediated modulation of presynaptic function. Moreover, through the screening of RNAi libraries, the MANTRA system has the potential to comprehensively identify novel genes and proteins involved in the regulation of neurotransmitter release.

The MANTRA system has major applications for the discovery of novel mechanism-based therapies for multiple central nervous system (CNS) disorders, many of which represent largely unmet medical needs. Alterations in presynaptic function have been implicated in schizophrenia [5], [6], depression [7], epilepsy [8], autism [9], attention deficit/hyperactivity disorder [10], Alzheimer's disease [11], Parkinson's disease [12], Huntington's disease [13], and migraine [14]. While significant progress has been made towards identifying the genetic and molecular alterations present in CNS disorders, it has been challenging to translate this body of knowledge into the identification of single, attractive drug targets that are amenable to traditional drug discovery efforts [41]. Rather, we propose that innovative, functional screening approaches are needed to address disease mechanisms for complex, multi-factorial psychiatric and neurological disorders. In this regard, the application of the MANTRA system to cellular models of CNS diseases may lead to the discovery of novel compounds or targets that restore aberrant synaptic function and serve as the basis for new mechanism-based treatments.

The repertoire of genetically encoded optical reporters for multiple neuronal processes is rapidly expanding [42]. Such reporters can be targeted to specific subcellular compartments by fusing them to proteins with appropriate targeting sequences [43], [44] and can be expressed in defined neuronal subtypes via cell-subtype specific promoters [45]. With the incorporation of such reporter systems, the applications of the MANTRA system can be expanded to address multiple aspects of neuronal function in different subcellular compartments of disease-relevant neuronal subtypes. With its unique and broad capacity for unbiased, high-throughput screening of synaptic function, we believe that the MANTRA system has the potential for major impact on basic neuroscience research and on CNS drug discovery.

## Materials and Methods

### Cell Culture

All experimental procedures were performed in accordance with the NIH Guide for the Care and Use of Laboratory Animals. In addition, all experimental procedures and protocols were reviewed and approved for use by the Galenea IACUC Committee. E18 embryos were recovered by postmortem caesarian section from euthanized pregnant Sprague-Dawley rats. Embryo forebrains were dissected, digested in HBSS (Invitrogen) containing 0.25% trypsin and 0.1% DNAse, and dissociated by trituration through fire-polished Pasteur pipettes. Neurons were plated and maintained in Neurobasal Medium (Invitrogen) plus 2% B-27 Supplement (Invitrogen), 500 µM glutamine (Invitrogen), and 6.25 µM glutamate (Sigma) and were incubated at 37°C in a 95% air/5% CO_2_ humidified incubator for 19–23 days before use. For high resolution assay experiments, neurons were plated onto poly-D-lysine (BD Biosciences) and laminin (BD Biosciences) coated 25-mm square coverslips (Carolina Biological Supply) inside a 5-mm-diameter cloning cylinder at 20,000 cells/cylinder. For the high-throughput assays, neurons were plated into poly-D-lysine coated, black-walled, thin-bottomed 96-well plates (Greiner Biosciences) at 75,000 cells/well in 150 µl/well of medium.

### Reporter Viral Transduction

The synaptophysin-pHluorin reporter [21] and the human synapsin promoter [23] sequences were as previously described. The expression construct was generated by custom cDNA synthesis (Blue Heron Bio). A recombinant adeno-associated virus of mixed serotype 1/2 (AAV1/2) was generated (GeneDetect). Titers of the viral preparations were >1X10^12^ GP/ml. At 7 DIV, neurons were infected with the hSyn-SypHy-AAV at 2500 MOI.

### Immunocytochemistry

Primary rat neuronal cultures on coverslips were infected with the hSyn-SypHy-AAV at 7 DIV. At 21 DIV, neurons were fixed in 4% formaldehyde, permeabilized in 0.3% Triton X-100, and blocked in 0.1% Triton X-100 plus 10% normal goat serum (Sigma). Neurons were incubated with primary antibodies, mouse anti-synaptotagmin (1∶1000; Synaptic Systems) and rabbit anti-GFP (1∶1000; Abcam), overnight at 4°C, rinsed in PBS, and incubated in secondary antibodies, Alexa-Fluor488 conjugated goat anti-rabbit IgG (1∶1000; Invitrogen) and Alexa-Fluor555 conjugated goat anti-mouse IgG (1∶1000; Invitrogen). Neurons were rinsed, mounted onto glass slides using Fluoromount G (Southern Biotech), and visualized with a Zeiss Axiovert Z1 microscope using a 40X 1.3NA oil immersion objective lens. Colocalization analysis was performed using ImageJ.

### High-resolution sypHy assays

Coverslips bearing neuronal cultures were washed in assay buffer which contained (in mM): NaCl 119, KCl 2.5, dextrose 30, HEPES 25, MgCl_2_ 2, CaCl_2_ 2, D-(−)-2-amino-5-phosphonopentanoic acid (D-AP5) 0.05, and 6,7-dinitroquinoxaline-2,3-dione (DNQX) 0.02. Coverslips were mounted in a custom-built perfusion and stimulation chamber secured to the stage of a Zeiss Axio Observer A1 microscope. Cultures were perfused at a rate of 100 µl/min using multiple peristaltic pumps, each connected to a different input reservoir. Pharmacological agents were introduced by switching peristaltic pumps. An objective warmer (Bioptechs) was used to maintain bath temperature at 30°C. To elicit action potentials, 1 ms voltage pulses (10 V) were passed between two 5 mm platinum sheet electrodes positioned on either side of the recording chamber. Stimulus patterns were delivered by a stimulus isolation unit (Coulbourn Instruments) controlled by Igor Pro software (Wavemetrics) and a DAQ system (National Instruments). Cultures were illuminated by a 475 nm LED (Cairn) and fluorescence was filtered with a 470/525 emission/excitation filter cube (Zeiss). Cultures were imaged with a 1.3 NA 40x oil-immersion objective lens, and fluorescence images were acquired with an iXON electron multiplying CCD (EMCCD) camera (Andor) with 50 msec exposures at a frequency of 1 Hz. Mean whole-field fluorescence intensity for each image was extracted using ImageJ. Resulting time-varying fluorescence waveforms were analyzed with custom routines (Igor Pro).

### MANTRA system assays

96-well plates containing neuronal cultures at 21–23 DIV were placed on the platform of an Evolution P3 liquid handling robot (EP3; Perkin Elmer) with which culture medium was replaced with assay buffer. Plates were transferred to a 30°C incubator for one hour, transferred to the plate tray in the MANTRA instrument, and subjected to a read/field stimulation protocol. Fluorescence readings were made using a 475/535 nm excitation/emission filter. Unless specified otherwise, field stimulus pulses were 30 V, 0.2 msec. This stimulation intensity was chosen for its ability to reliably initiate action potentials in all neurons in all wells. SypHy responses to these stimuli were completely abolished by TTX, demonstrating that these responses are action potential-mediated (see Figure 4A). The temperature of the cabinet was set at 32°C, resulting in an assay buffer temperature of 30.5 to 31.5°C. Wells were imaged at 1 Hz with 100 msec exposures. Data files were post-processed using in-house analysis routines (Igor Pro) and stored in a custom MySQL database. As a result of sporadic inconsistencies in assay buffer aspiration into the central capillary of the electrodes, stimulation failures can occasionally occur. Failures were specified as any wells in which the ΔF/F in response to a 50 Hz, 15 sec or a 30 Hz, 10 sec stimulation train was less than 0.05. Typically 0–3 wells were removed from analyses from each 96-well plate as a result of stimulation failures.

For compound screening, a 1.5 mM dilution plate was generated using an EP3 liquid handling robot (Perkin Elmer). 2 µl of compounds were transferred from this dilution plate to each of three near assay-ready compound plates in pseudo-randomized locations using a Janus liquid handling station (Perkin Elmer). Assays were performed as described above. Compounds, including TTX (Tocris) and the Ca^++^ channel inhibitors ω-agatoxin IVA, ω-contoxin GVIA, and SNX-482 (Alomone Labs), were added with the final buffer addition of the plate wash process.

## Supporting Information

Figure S1 MANTRA system temperature control system validation. Well temperature was measured using a thermocouple inserted into well H1 of a 96-well plate during an electrostimulation protocol. Temperature of the metal plate tray, monitored by an independent thermocouple inserted into the tray, remained constant at 32°C throughout the run. The plate was preincubated at 31°C and placed on the plate tray in the instrument. The plate lid was removed and temperature logging was started at t = 0. Removal of the lid caused the temperature to drop due to evaporation. Within one minute the tip module entered the plate wells. The presence of the tip module reduced evaporation causing the temperature to re-equilibrate to approximately 31°C. When the tip module was removed at the end of the run a temperature decrease was again observed. Well temperature remained within 0.5°C of the target temperature of 31°C throughout the 35 minute stimulation protocol.
(TIF)

Figure S2 Confirmation of hits from the LOPAC library plate on the MANTRA system. Three assay plates were run on the MANTRA instrument each containing six replicates (10 µM) of each of the twelve hit compounds from LOPAC plate 13 (n = 18; see Figure 5C). Shown are the amplitudes of the responses to 5 Hz stimulation (mean ± SEM) normalized to the mean amplitude of the eight vehicle wells on the same plate. Each compound altered the response amplitude in the direction observed on the initial screening plates (t-test; ** p<0.001; *** p<0.00001).
(TIF)

Figure S3 Response amplitudes for compounds in a plate of adenosine/purinergic-focused compounds. A plate containing compounds targeting purine and adenosine receptors (Biomol) was screened as described in Figure 6. Shown are the amplitudes of the responses to the 5 Hz (**A**) and 30 Hz (**B**) trains normalized to the vehicle controls. Dotted lines indicate three standard deviations from the mean of vehicle wells. Green circles indicate hit compounds that increases the 30 Hz:5 Hz response amplitude ratio (see Figure 6).
(TIF)

Table S1 Summary statistics from 8 control plates for 5 Hz, 30 sec trains. See **Table 1** for explanation. Baseline noise is as in Table 1.
(DOC)

Table S2 Summary statistics from 8 control plates for 50 Hz, 15 sec trains. See **Table 1** for explanation. Baseline noise is as in Table 1.
(DOC)

Dataset S1 MANTRA system screening data for a single LOPAC plate.
(XLS)

Dataset S2 MANTRA system screening data for adenosine receptor compound plate.
(XLS)
