# Supplementary figures and images for: A System for Performing High Throughput Assays of Synaptic Function
Source: PLoS One. 2011 Oct 5;6(10):e25999. doi: 10.1371/journal.pone.0025999 (PMC3187845; doi:10.1371/journal.pone.0025999)

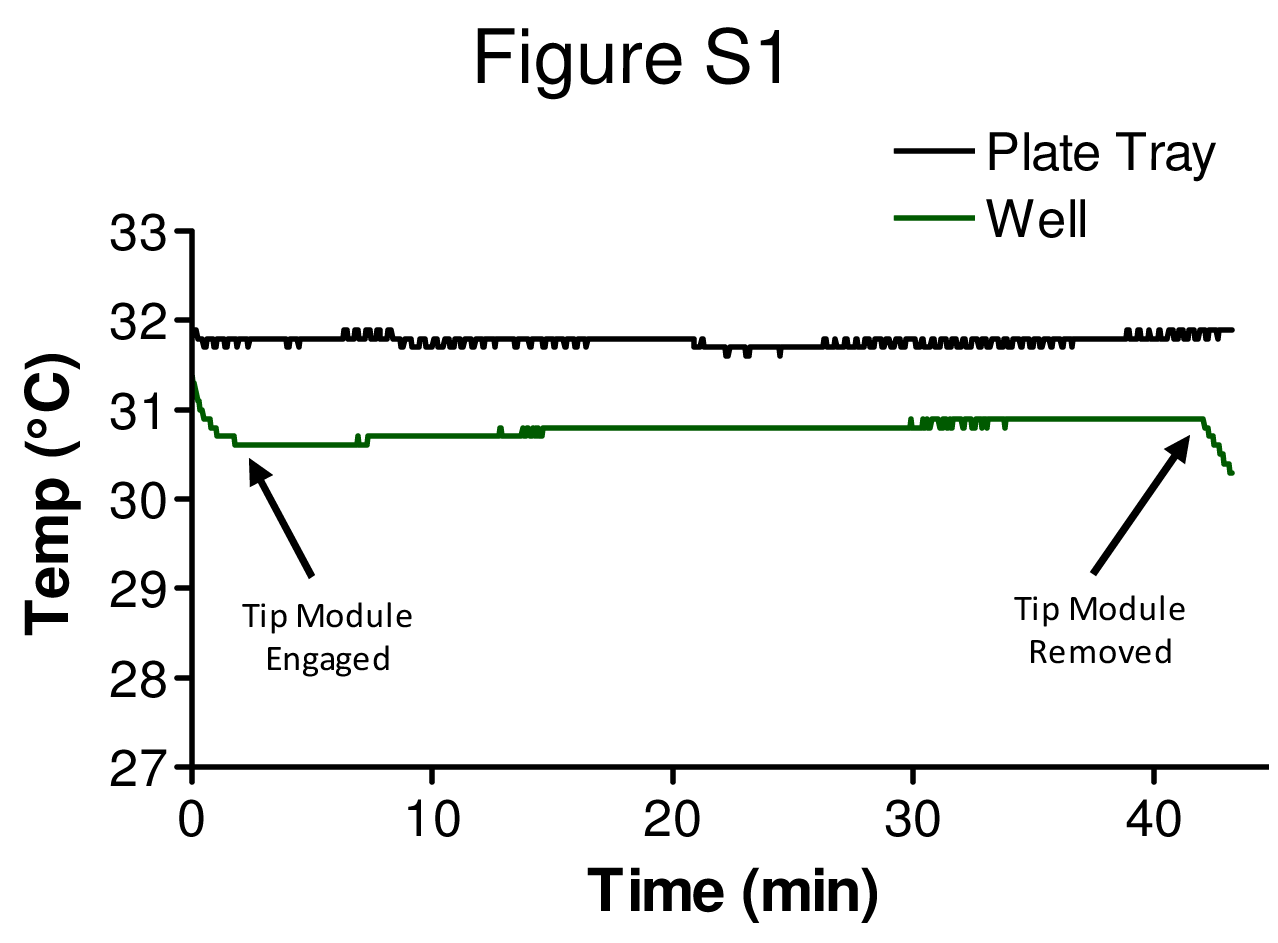

Supplement: Figure S1 — MANTRA system temperature control system validation. Well temperature was measured using a thermocouple inserted into well H1 of a 96-well plate during an electrostimulation protocol. Temperature of the metal plate tray, monitored by an independent thermocouple inserted into the tray, remained constant at 32°C throughout the run. The plate was preincubated at 31°C and placed on the plate tray in the instrument. The plate lid was removed and temperature logging was started at t = 0. Removal of the lid caused the temperature to drop due to evaporation. Within one minute the tip module entered the plate wells. The presence of the tip module reduced evaporation causing the temperature to re-equilibrate to approximately 31°C. When the tip module was removed at the end of the run a temperature decrease was again observed. Well temperature remained within 0.5°C of the target temperature of 31°C throughout the 35 minute stimulation protocol. (TIF) [file pone.0025999.s001.tif]

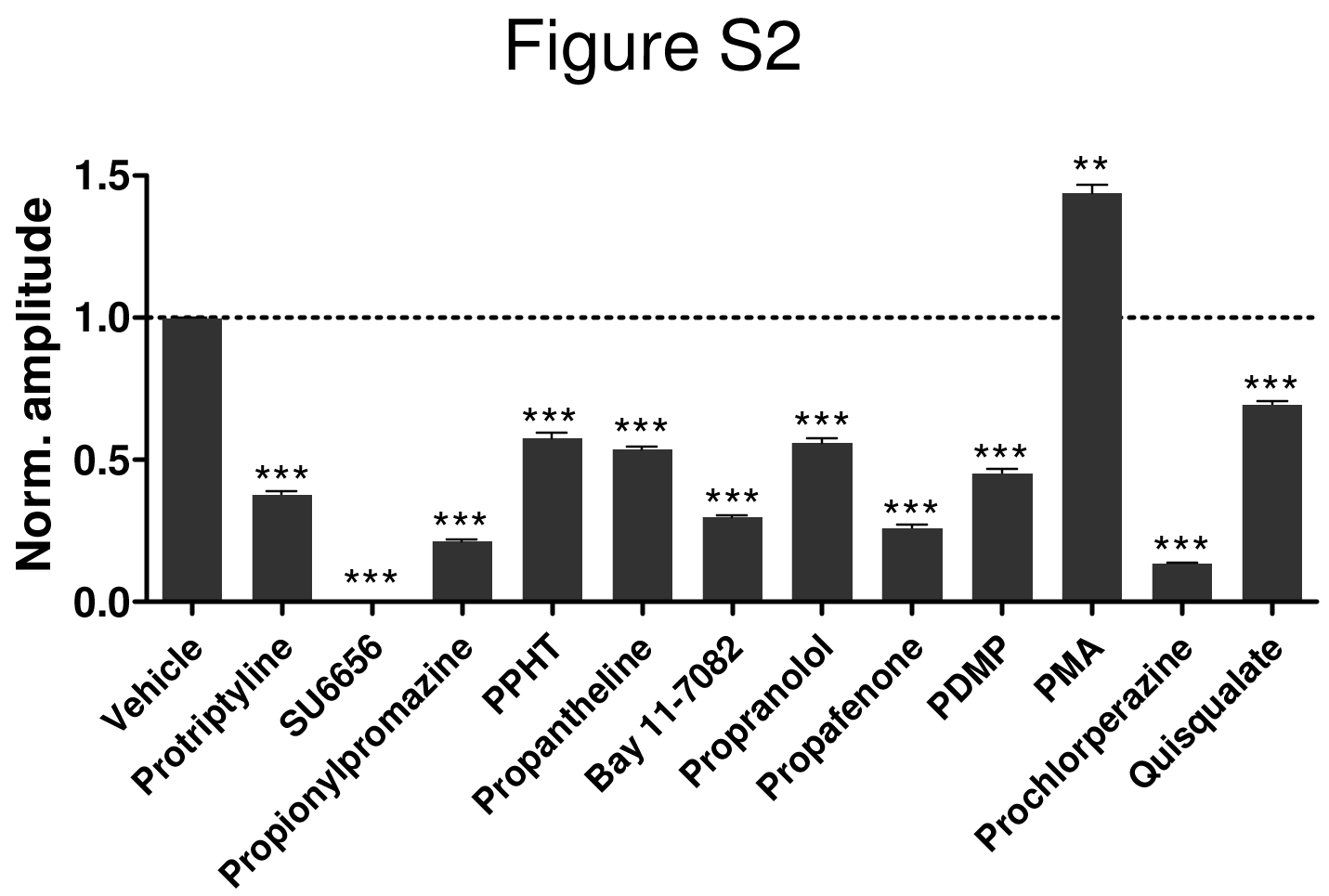

Supplement: Figure S2 — Confirmation of hits from the LOPAC library plate on the MANTRA system. Three assay plates were run on the MANTRA instrument each containing six replicates (10 µM) of each of the twelve hit compounds from LOPAC plate 13 (n = 18; see Figure 5C). Shown are the amplitudes of the responses to 5 Hz stimulation (mean ± SEM) normalized to the mean amplitude of the eight vehicle wells on the same plate. Each compound altered the response amplitude in the direction observed on the initial screening plates (t-test; ** p<0.001; *** p<0.00001). (TIF) [file pone.0025999.s002.tif]

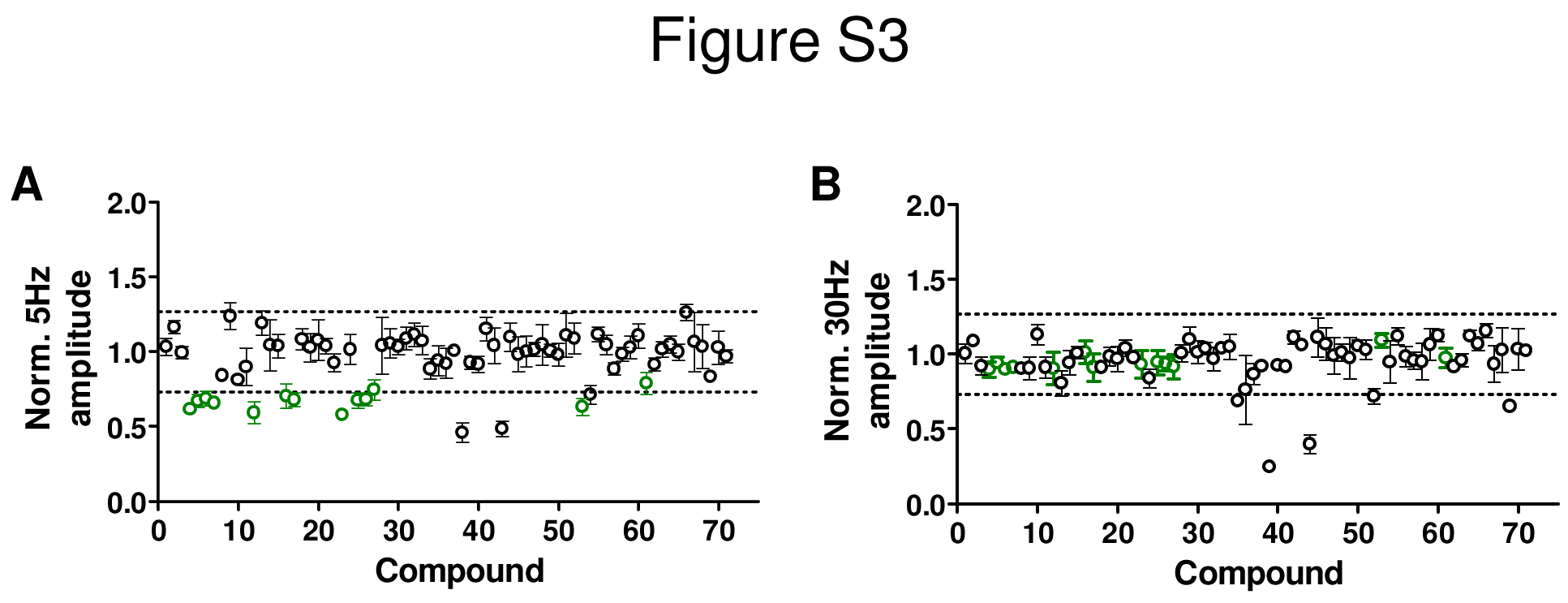

Supplement: Figure S3 — Response amplitudes for compounds in a plate of adenosine/purinergic-focused compounds. A plate containing compounds targeting purine and adenosine receptors (Biomol) was screened as described in Figure 6. Shown are the amplitudes of the responses to the 5 Hz (A) and 30 Hz (B) trains normalized to the vehicle controls. Dotted lines indicate three standard deviations from the mean of vehicle wells. Green circles indicate hit compounds that increases the 30 Hz:5 Hz response amplitude ratio (see Figure 6). (TIF) [file pone.0025999.s003.tif]
