# Supplementary material for: A System for Performing High Throughput Assays of Synaptic Function
Source: PLoS One. 2011 Oct 5;6(10):e25999. doi: 10.1371/journal.pone.0025999 (PMC3187845; doi:10.1371/journal.pone.0025999)
Supplement: Table S1 — Summary statistics from 8 control plates for 5 Hz, 30 sec trains. See Table 1 for explanation. Baseline noise is as in Table 1. (DOC) [file pone.0025999.s004.doc]

|  |  | **Individual Plates** | | | | | | | | **All Plates** | | |
| --- | --- | --- | --- | --- | --- | --- | --- | --- | --- | --- | --- | --- |
| **Parameter** |  | **1** | **2** | **3** | **4** | **5** | **6** | **7** | **8** | **Mean** | **SD** | **%CV** |
| **Amplitude**  **(ΔF/F)** | Mean | 0.09 | 0.08 | 0.08 | 0.10 | 0.08 | 0.09 | 0.09 | 0.10 | 0.09 | 0.01 | 8.0 |
|  | SD | 0.01 | 0.01 | 0.01 | 0.01 | 0.01 | 0.01 | 0.01 | 0.01 |  |  |  |
|  | %CV | 11.6 | 11.0 | 10.9 | 9.9 | 13.8 | 15.0 | 11.6 | 12.5 | 12.0 |  |  |
| **Decay τ**  **(sec)** | Mean | 16.7 | 14.1 | 14.7 | 16.6 | 17.3 | 19.3 | 18.0 | 19.9 | 17.1 | 2.02 | 11.8 |
|  | SD | 1.66 | 1.92 | 1.80 | 2.07 | 2.59 | 2.87 | 2.68 | 2.13 |  |  |  |
|  | %CV | 9.9 | 13.6 | 12.3 | 12.5 | 14.9 | 14.9 | 14.9 | 10.7 | 13.0 |  |  |
| **Derivative**  **(ΔF/sec)** | Mean | 0.015 | 0.014 | 0.015 | 0.015 | 0.011 | 0.017 | 0.016 | 0.015 | 0.015 | 0.002 | 12.6 |
|  | SD | 0.003 | 0.003 | 0.003 | 0.002 | 0.002 | 0.004 | 0.004 | 0.002 |  |  |  |
|  | %CV | 17.9 | 20.0 | 20.2 | 17.0 | 16.4 | 20.8 | 23.2 | 17.0 | 19.1 |  |  |
| **Integral**  **(ΔF/F x sec)** | Mean | 1.80 | 1.64 | 1.73 | 2.13 | 1.81 | 1.78 | 1.88 | 1.99 | 1.85 | 0.15 | 8.3 |
|  | SD | 0.23 | 0.21 | 0.20 | 0.24 | 0.26 | 0.29 | 0.21 | 0.27 |  |  |  |
|  | %CV | 12.7 | 12.8 | 11.8 | 11.3 | 14.5 | 16.2 | 11.4 | 13.6 | 13.0 |  |  |
|  |  |  |  |  |  |  |  |  |  |  |  |  |

**Table S1.** MANTRA system signal uniformity analysis. Eight control plates were subjected to a stimulus protocol comprised of 1) a 5 Hz, 30 sec, 2) a 10 Hz, 30 sec, and 3) a 50 Hz, 15 sec pulse train in succession, with a 5 minute inter-train interval. Amplitude, decay time constant, peak first derivative, and response integral for the response to the 5 Hz train are shown.
