# Supplementary material for: A System for Performing High Throughput Assays of Synaptic Function
Source: PLoS One. 2011 Oct 5;6(10):e25999. doi: 10.1371/journal.pone.0025999 (PMC3187845; doi:10.1371/journal.pone.0025999)
Supplement: Table S2 — Summary statistics from 8 control plates for 50 Hz, 15 sec trains. See Table 1 for explanation. Baseline noise is as in Table 1. (DOC) [file pone.0025999.s005.doc]

|  |  | **Individual Plates** | | | | | | | | **All Plates** | | |
| --- | --- | --- | --- | --- | --- | --- | --- | --- | --- | --- | --- | --- |
| **Parameter** |  | **1** | **2** | **3** | **4** | **5** | **6** | **7** | **8** | **Mean** | **SD** | **%CV** |
| **Amplitude**  **(ΔF/F)** | Mean | 0.22 | 0.26 | 0.25 | 0.28 | 0.21 | 0.26 | 0.24 | 0.23 | 0.24 | 0.02 | 9.5 |
|  | SD | 0.04 | 0.05 | 0.04 | 0.03 | 0.04 | 0.05 | 0.05 | 0.05 |  |  |  |
|  | %CV | 17.9 | 17.5 | 16.0 | 11.7 | 19.2 | 17.5 | 18.9 | 19.6 | 17.3 |  |  |
| **Decay τ**  **(sec)** | Mean | 20.2 | 18.5 | 17.8 | 21.7 | 17.3 | 23.4 | 21.7 | 21.1 | 20.2 | 2.17 | 10.7 |
|  | SD | 2.07 | 2.18 | 2.25 | 2.51 | 1.82 | 3.89 | 2.72 | 2.46 |  |  |  |
|  | %CV | 10.2 | 11.8 | 12.7 | 11.6 | 10.5 | 16.6 | 12.5 | 11.6 | 12.2 |  |  |
| **Derivative**  **(ΔF/sec)** | Mean | 0.038 | 0.041 | 0.043 | 0.048 | 0.039 | 0.043 | 0.041 | 0.040 | 0.042 | 0.003 | 7.6 |
|  | SD | 0.006 | 0.007 | 0.007 | 0.006 | 0.007 | 0.007 | 0.007 | 0.007 |  |  |  |
|  | %CV | 15.9 | 16.8 | 15.2 | 13.2 | 17.0 | 17.4 | 16.9 | 18.0 | 16.3 |  |  |
| **Integral**  **(ΔF/F x sec)** | Mean | 2.52 | 3.06 | 2.80 | 3.23 | 2.66 | 2.89 | 2.86 | 2.80 | 2.85 | 0.219 | 7.7 |
|  | SD | 0.43 | 0.53 | 0.45 | 0.38 | 0.51 | 0.50 | 0.53 | 0.55 |  |  |  |
|  | %CV | 17.2 | 17.5 | 15.9 | 11.6 | 19.3 | 17.4 | 18.5 | 19.5 | 17.1 |  |  |
|  |  |  |  |  |  |  |  |  |  |  |  |  |

**Table S2.** MANTRA system signal uniformity analysis. Eight control plates were subjected to a stimulus protocol comprised of 1) a 5 Hz, 30 sec, 2) a 10 Hz, 30 sec, and 3) a 50 Hz, 15 sec pulse train in succession, with a 5 minute inter-train interval. Amplitude, decay time constant, peak first derivative, and response integral for the response to the 50 Hz train are shown.
